# Nebulized Versus Intravenously Administered Dexmedetomidine for Obtunding Hemodynamic Responses to Laryngoscopy and Tracheal Intubation: A Randomized Double-Blind Comparative Study

**DOI:** 10.7759/cureus.54768

**Published:** 2024-02-23

**Authors:** Akshita Singla, Rajkumar K Saraswat, Avnish Bharadwaj, Sapna Singh

**Affiliations:** 1 Anaesthesiology, Mahatma Gandhi Medical College and Research Institute, Jaipur, IND

**Keywords:** hemodynamic response, nebulization, intravenous dexmedetomidine, intubation, laryngoscopy

## Abstract

Introduction

Intravenous dexmedetomidine is known to cause major adverse effects such as bradycardia, hypotension, cardiac arrhythmias, and heart block when used as premedication for attenuation of the laryngoscopy and intubation response, limiting its routine use. Thus, it is important to study other routes of administration of dexmedetomidine.

Objectives

To compare the hemodynamic response and sedation score between intravenous and nebulized dexmedetomidine as premedication for the attenuation of the laryngoscopy and intubation response.

Materials and methods

In this study, 60 patients fulfilling inclusion criteria undergoing surgeries under general anesthesia (ASA Grade I and II) were randomly allocated into two groups of 30 patients each. Group IV received intravenous 1 mcg/kg dexmedetomidine in 100 mL normal saline, and Group IN received nebulization with 1 mcg/kg dexmedetomidine diluted to a total volume of 5 cc of normal saline, 30 minutes prior to the induction of general anesthesia. Sedation scores were calculated using the Ramsay sedation score at 20 minutes after the administration of the drug; patients were induced by the standard protocol, and laryngoscopy was performed. Vitals were recorded before the administration of the drug and after intubation at stipulated time intervals.

Results

The median heart rate becomes significantly lower at 15 minutes (70 vs. 76.5) and 20 minutes (66 vs. 76) after induction among Group IV as compared to Group IN. The median systolic blood pressure was significantly lower at 20 minutes in Group IV (110 mmHg) than in Group IN (119 mmHg). The median diastolic blood pressure was significantly lower at 10 minutes (76 vs. 79), 15 minutes (70 vs. 77), and 20 minutes (69 vs. 78.5) in Group IV than in Group IN. The median of mean arterial pressure was significantly lower at 15 minutes (84.8 vs. 91.5) and 20 minutes (83 vs. 92) in Group IV than in Group IN. A comparison of vitals after induction shows that the median heart rate, systolic blood pressure, diastolic blood pressure, and mean arterial pressure were significantly lower statistically among Group IV as compared to Group IN at 0, 1, 3, 5, 10, 15, and 30 minutes after induction (except for systolic blood pressure at 3 minutes). The median sedation score was lower in Group IN (0) than in Group IV (1); this difference is statistically significant.

Conclusion

The obtundation of hemodynamic responses following laryngoscopy and maintaining hemodynamics intraoperatively is statistically better with nebulized dexmedetomidine compared to intravenous dexmedetomidine.

## Introduction

Laryngoscopy and tracheal intubation are common procedures under anesthesia that often elicit significant hemodynamic responses, including hypertension and tachycardia. These responses can pose challenges, particularly in patients with cardiovascular comorbidities. Laryngoscopy, tracheal intubation, surgical stimulation, extubation, and other airway instrumentations incite remarkable sympathetic activity and cause an intense noxious stimulus through vagal afferents and glossopharyngeal afferents that result in transient but significant hemodynamic changes, termed as pressor/hemodynamic stress response due to the reflex activation of the autonomic system. To avoid such complications and consequences, various drugs are used for attenuation of the laryngoscopic response. α-2 agonists such as dexmedetomidine are one such class of drugs [1-2].

Dexmedetomidine is a potent and highly selective alpha-2 receptor agonist with sympatholytic, sedative, amnestic, and analgesic properties [3]. Its pleiotropic effects have led to its increasing use for reducing anesthetic and analgesic requirements in the perioperative period [3]. The efficacy of dexmedetomidine in decreasing the hemodynamic response to laryngoscopy and intubation has been studied using intravenous [4-8], intranasal [9,10], and intramuscular routes [11]. Intravenous administration is the most common route for the administration of dexmedetomidine. However, intravenous administration may cause bradycardia, hypotension, cardiac arrhythmias, and heart block. Intranasal administration may be associated with irritation [12].

Dexmedetomidine can also be administered via intranasal, nebulization, oral, intramuscular, and intrathecal routes [9,13-15]. Thus, it is important to study other routes of administration.

Nebulized dexmedetomidine, administered in doses of 1 and 2 µg/kg, has been found to be an effective premedication in pediatric patients [11,12]. Nebulized dexmedetomidine may offer an attractive alternative to both intravenous and intranasal routes of administration because drug deposition following nebulization takes place over nasal, buccal, as well as respiratory mucosa [11,12], resulting in better systemic absorption and thus achieving the attenuation of laryngoscopy response, sedation, and analgesia effects [13-15]. Thus, alternative modes of dexmedetomidine administration are expected to limit the adverse effects and enhance the safety profile of dexmedetomidine [14]. The efficacy of intranasal dexmedetomidine has been investigated with established safety, efficacy, and high patient acceptance [15].

This randomized double-blind comparative study aimed to investigate the comparative effectiveness of nebulized dexmedetomidine versus the traditional intravenous route in attenuating hemodynamic responses associated with laryngoscopy and tracheal intubation. As the demand for optimized anesthetic techniques continues to grow, exploring novel administration methods for dexmedetomidine holds promise for improving perioperative outcomes and enhancing patient safety. This research addresses a critical gap in the current literature, providing valuable insights into the potential benefits of nebulized dexmedetomidine in the context of airway manipulation during surgery.

The primary objective was to compare nebulized dexmedetomidine with intravenous dexmedetomidine as premedication for its effect on heart rate, systolic blood pressure (BP), diastolic BP, and mean arterial pressure (MAP) following laryngoscopy and tracheal intubation. The secondary objective was to study and compare the sedation score and to study and compare the adverse effects encountered with the above two routes of administration.

## Materials and methods

This study was conducted after obtaining approval from the Institutional Ethics Committee, Mahatma Gandhi Medical College and Research Institute, Jaipur (letter number: 1676). This trial was registered on the Clinical Trials Registry, India (CTRI/2023/10/058557).

Inclusion criteria

This is a hospital-based randomized double-blind prospective comparative study. After obtaining informed written consent, 60 patients with the American Society of Anesthesiologists grade I and II of either sex, aged 18-55 years, and with a body weight of 50-70 kg undergoing elective surgery under general endotracheal anesthesia were included in the study.

Exclusion criteria

Patient refusal for the anesthetic technique, patients with severe renal, hepatic, respiratory, or cardiac diseases, bleeding disorders, or those on anticoagulants, history of allergy to the study drug, patients on any sedatives, antipsychotics, antidepressants, anxiolytics, or anticonvulsants and beta-blockers, patients with any nasal pathology such as nasal ulcers, nasal polyps, or nasal septum deviation, patients with difficult airways like cervical spine instability, facial fractures, partially obstructing laryngeal lesions (e.g., papilloma), craniofacial abnormalities, and temporomandibular joint ankylosis, patients coming for emergency surgery, pregnant patients, and history of COVID infection during the last three months were excluded.

 

Sample size calculation

The objective of this study is to compare nebulized versus intravenously administered dexmedetomidine for obtunding hemodynamic responses to laryngoscopy and intubation. One of the indicators for hemodynamic response is the heart rate after intubation. According to a similar study conducted by Suryawanshi et al., the mean heart rate in the control group at 10 minutes after induction was 88 per minute, and the standard deviation (SD) was 8.11, while the mean heart rate in the intervention group was 81 per minute with an SD of 8.82 [1]. So, for sample size calculation considering the above parameters and taking alpha as 0.05 to have a 95% confidence level with the power of the study (beta) as 90%, we used the following formula to calculate the final sample size: N = 2SD^2^ (Z1+Z2)^2^ / (M1-M2)^2^, where "SD" represents the pooled standard deviation, calculated as 8.47; "Z1" is the Z-value associated with alpha, set at 1.64; "Z2" is the Z-value associated with beta, set at 1.28; "M1" is the mean heart rate in the intervention group, which is 88; and "M2" is the mean heart rate in the control group, which is 81. Using the above factors, we arrived at a sample size of 26 in each group. We considered an additional 10% of the sample to compensate for incomplete data or non-response. So, the final sample size N = 26 + (10% of 26) = 26 + 2.6 = 28.6. Making it a round number, N = 30 in each group.

Method

After a thorough pre-anesthetic check-up a day before, patients were shifted to the preoperative holding area, and randomization was done for group allocation by computer-generated random numbers. Standard ASA monitors such as electrocardiogram, noninvasive BP, and pulse oximeter were attached. Group IV (n=30) received 1 mcg/kg dexmedetomidine given in 100 mL of normal saline intravenously 30 minutes prior to induction of general anesthesia over 15 minutes. Simultaneously, they were nebulized with 5 mL of normal saline. Group IN (n=30) was nebulized with 1 mcg/kg dexmedetomidine diluted to a total volume of 5 mL with normal saline and simultaneously received 100 mL of normal saline intravenously 30 minutes prior to induction of general anesthesia over 15 minutes. Vital parameters were monitored every 5 minutes. Ramsay sedation scores were recorded at 20 minutes, wherein 0 means the patient is awake and following verbal commands, 1 means the patient is drowsy but following verbal commands, 2 means the patient is drowsy responding to touch, 3 means the patient is drowsy responding to noxious stimuli, and 4 means the patient is unresponsive to noxious stimuli (Table 1).

**Table 1:** Ramsay sedation scores

Clinical score	Patient characteristics
0	Patient awake and following verbal commands
1	Patient drowsy but following verbal commands
2	Patient drowsy responding to touch
3	Patient drowsy responding to noxious stimuli
4	Patient unresponsive to noxious stimuli

Technique of anesthesia

Patients were shifted to the operating room, breathing two liters of oxygen per minute through a Hudson mask. Anesthesia was induced following a standard protocol. Patients were premedicated with IV glycopyrrolate 0.004 mg/kg and IV butorphanol 0.02 mg/kg, and induction was achieved with IV propofol 1.5 mg/kg. Muscle relaxation was obtained with 2 mg/kg of succinylcholine. Laryngoscopy was performed at the end of one minute after administering succinylcholine, and patients were intubated using an appropriately sized endotracheal tube. Heart rate, systolic BP, diastolic BP, mean arterial pressure (MAP), and pulse oximetry were recorded upon starting the study drugs and after every five-minute interval until the endotracheal tube was inserted. Additionally, vitals were recorded immediately after intubation, at 1, 3, and 5 minutes after intubation, and thereafter every 5 minutes up to 30 minutes after the insertion of the endotracheal tube. Adverse effects, such as bradycardia, were noted and treated with 0.01 mg/kg atropine IV. Hypotension was treated with crystalloids and an injection of mephentermine 3 to 6 mg IV.

Analysis

Statistical analysis was carried out using Microsoft Excel 2013 (Microsoft Corporation, Redmond, Washington) and IBM SPSS Statistics for Windows, Version 21 (Released 2012; IBM Corp., Armonk, New York). Chi-square, Mann-Whitney U, and independent t-tests were applied depending on the pattern of distribution of the data, and a P-value < 0.05 was considered significant. All data were first analyzed for normal distribution by Kolmogorov-Smirnov and Shapiro-Wilk tests. The mean value with a parametric test (independent t-test) was applied to data that followed a normal distribution, while the median value with non-parametric tests (Mann-Whitney U test) was applied to data that did not follow a normal distribution.

## Results

The median age, weight, and height were not significantly different between the two groups, as suggested by the Mann-Whitney U test, indicating that the baseline characteristics of patients in both groups were comparable (Table 2). Comorbidities like diabetes mellitus (DM) and hypertension (HT) were not significantly different between the two groups, as revealed by Fisher's exact test (Table 3).

**Table 2:** Comparison of basic parameters between the two groups (N=60) Group IV: Intravenous group; Group IN: Nebulized group; IQR: interquartile range.

Basic parameters	Group IV (n=30)		Group IN (n=30)		Mann-Whitney U test	
	Median	IQR	Median	IQR	Z-value	P-value
Age (years)	40	26	42.5	18	0.8	0.42
Weight (kg)	67	20	62.5	14	1.21	0.22
Height (cm)	160	10	162.5	11	1.17	0.24

**Table 3:** Comparison of comorbidities between the two groups (N= 60) Group IV: Intravenous group; Group IN: Nebulized group.

Comorbidities		Group IV (n=30)		Group IN (n=30)		Fisher’s exact test
		No.	%	No.	%	P-value
Diabetes	Yes	0	0	2	6.7	0.49
	No	30	100	28	93.3	
Hypertension	Yes	01	3.3	03	10	0.61
	No	29	96.7	27	90	
Others	Yes	00	0	03	10	0.11
	No	30	100	27	90	

There was no statistically significant difference in the American Society of Anesthesiologists (ASA) grade between the two groups. Group IV included 93.3% ASA grade 1 patients and 6.7% ASA grade 2 patients, while Group IN included 80% ASA grade 1 patients and 20% ASA grade 2 patients (Table 4). The baseline comparison of vitals between the two groups showed no statistically significant difference in heart rate, systolic BP, diastolic BP, MAP, respiratory rate, and temperature (Table 5).

**Table 4:** Comparison of ASA grade between the two groups (N=60) ASA: American Society of Anesthesiologists; Group IV: Intravenous group; Group IN: Nebulized group.

ASA grade	Group IV		Group IN		Fisher’s exact test
	No.	%	No.	%	P-value
ASA-1	28	93.3	24	80	0.25
ASA-2	02	6.7	06	20	

**Table 5:** Comparison of vitals between the two groups (N=60) Group IV: Intravenous group; Group IN: Nebulized group.

Baseline vitals	Group IV (n=30)		Group IN (n=30)		Mann-Whitney U test	
	Median	IQR	Median	IQR	Z-value	P-value
Heart rate	83	11	80	10	1.2	0.22
Systolic BP	128	12	128	14	1.1	0.26
Diastolic BP	83	10	80	11	1.03	0.3
Mean arterial pressure (MAP)	98.7	8.6	96	12.5	1.22	0.32
Respirate rate/minute	14	02	14	01	0.31	0.75
Body temperature (ºF)	98.6	0.3	98.6	0.1	1.82	0.06

**Figure 1:**
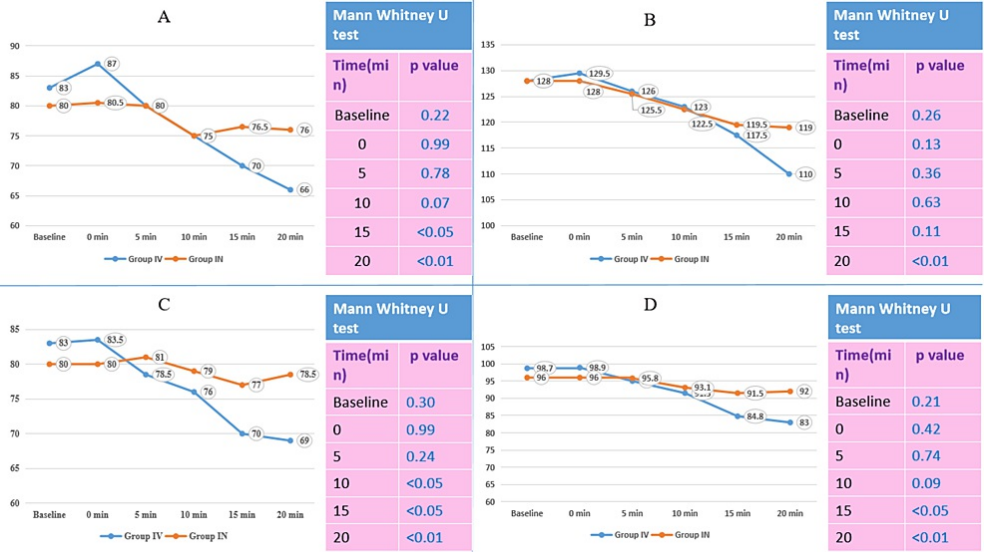
Comparison of vitals before induction A: Comparison of heart rate before induction; B: comparison of systolic blood pressure before induction; C: comparison of diastolic blood pressure before induction; D: comparison of mean arterial pressure before induction. Group IV: Intravenous group; Group IN: Nebulized group.

The median heart rate at baseline, 0 minutes, 5 minutes, and 10 minutes after induction were not found significantly different. However, the median heart rate became significantly lower at 15 minutes (70 vs. 76.5) and 20 minutes (66 vs. 76) after induction in Group IV compared to Group IN. The median systolic BP was significantly lower at 20 minutes in Group IV (110 mmHg) than in Group IN (119 mmHg). The median diastolic BP was significantly lower at 10 minutes (76 vs. 79), 15 minutes (70 vs. 77), and 20 minutes (69 vs. 78.5) in Group IV than in Group IN. The median of MAP was significantly lower at 15 minutes (84.8 vs. 91.5) and 20 minutes (83 vs. 92) in Group IV than in Group IN (Figure 1).

In Group IV, 2 out of 30 patients experienced bradycardia and hypotension at 10 minutes and 15 minutes, leading to the cessation of drug administration in both patients. One of these patients required 6 mg IV mephentermine for the correction of hypotension. As more than half the volume of the drug had already been delivered, these cases were included in the study.

In Group IN, one patient developed significant tachycardia 5 minutes after the completion of nebulization, which persisted throughout the surgery.

**Figure 2:**
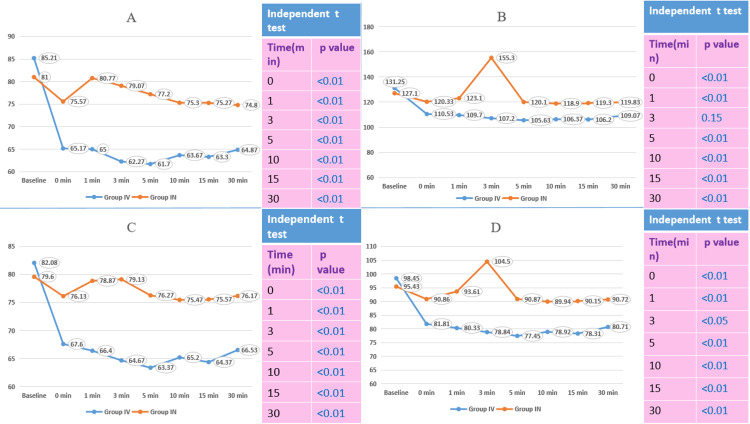
Comparison of vitals after induction A: Comparison of heart rate after induction; B: comparison of systolic blood pressure after induction; C: comparison of diastolic blood pressure after induction; D: comparison of mean arterial pressure after induction. Group IV: Intravenous group; Group IN: Nebulized group.

The comparison of vitals after induction showed that the median heart rate, systolic BP, diastolic BP, and MAP were statistically significantly lower in Group IV compared to Group IN at 0, 1, 3, 5, 10, 15, and 30 minutes after induction (except systolic BP at 3 minutes) (Figure 2).

**Figure 3:**
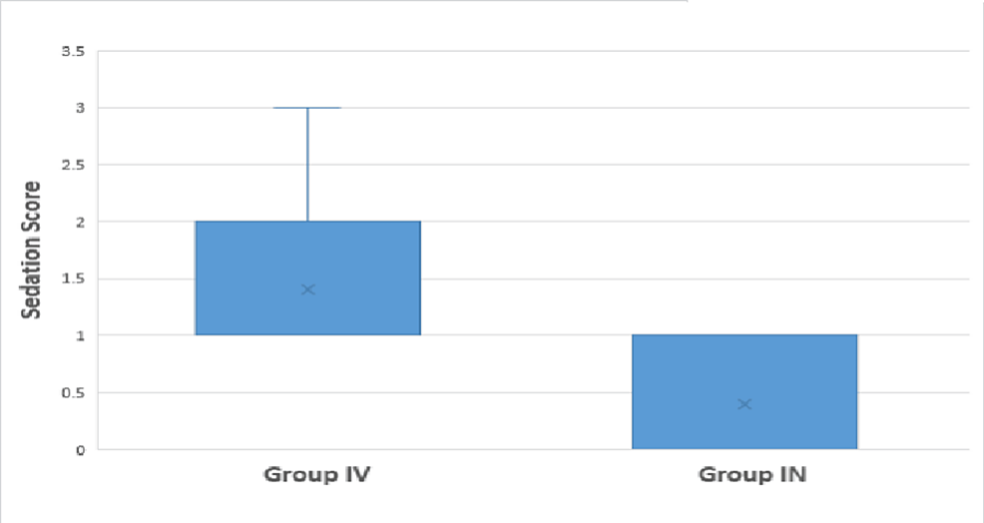
Comparison of sedation score between both groups Group IV: Intravenous group; Group IN: Nebulized group.

The median sedation score was lower in Group IN (0) than in Group IV (1), and this difference was statistically significant (Mann-Whitney U test Z=5.45, P-value <0.01) (Figure 3).

The comparison of hemodynamic responses between both groups showed that the differences in vitals between 0 and 1 minute and 0 and 3 minutes between Group IV and Group IN were statistically significant (Table 6).

**Table 6:** Comparison of obtundation of hemodynamic responses between the two groups (N=60) Group IV: Intravenous group; Group IN: Nebulized group; IQR: interquartile range.

Vitals	Difference between 0 and 1 minute vitals					Difference between 0 and 3 minute vitals				
	Group IV		Group IN		P-value	Group IV		Group IN		P-value
	Median	IQR	Median	IQR		Median	IQR	Median	IQR	
Heart rate	-1	4	3.5	10	<0.01	-2.5	4	1	11	<0.01
Systolic blood pressure	-1	3	3	7	<0.01	-4	5	1	10	<0.01
Diastolic blood pressure	-2	3	2.5	10	<0.01	-3	7	2	12	<0.01
Mean arterial pressure	-1.5	4.08	2.33	7.75	<0.01	-3	5.6	2.3	10.75	<0.01

There was no significant hemodynamic change statistically during laryngoscopy. However, with intravenous dexmedetomidine, there was a significant change in hemodynamics before laryngoscopy, indicating that the nebulized form did not lead to major adverse effects such as bradycardia, hypotension, and maintained the vitals throughout the observation period.

The obtundation of the laryngoscopic response was observed in both groups, but the hemodynamic response was better with the nebulized route.

## Discussion

In this prospective, randomized, double-blind, comparative study, hemodynamic responses following laryngoscopy and tracheal intubation were more favorable with nebulized dexmedetomidine. Intravenously administered dexmedetomidine resulted in a greater decrease in heart rate, systolic and diastolic BPs, and MAP, both before and after induction of anesthesia compared to dexmedetomidine administered by the nebulized route. The sedation score was lower with nebulized dexmedetomidine compared to intravenous administration.

The comparison of basic parameters between Group IV (intravenously administered dexmedetomidine) and Group IN (nebulized dexmedetomidine) in this study did not reveal any significant differences in age, weight, or height, and comorbidities. The lack of significant differences in age, weight, and height between the two groups suggests that the randomization process was effective in creating clinically homogeneous groups. The absence of significant differences in the distribution of comorbidities enhances the internal validity of the study. This is essential for isolating the effects of the mode of dexmedetomidine administration on hemodynamic responses during laryngoscopy and tracheal intubation. This strengthens the internal validity of the study, enhancing the confidence in attributing the outcomes to the mode of dexmedetomidine delivery. A similar study conducted by Singh et al. [16] and Ankita et al. [17] also shows that age, BMI, and gender are comparable. The results of this baseline comparison pave the way for a focused analysis of the primary study outcomes and enhance the robustness of the study.

The comparison of comorbidities between patients receiving intravenously administered dexmedetomidine (Group IV) and nebulized dexmedetomidine (Group IN) in this study provides valuable insights into the potential influence of baseline health conditions on hemodynamic responses during laryngoscopy and tracheal intubation. It suggests that any observed differences in hemodynamic responses during airway manipulation are more likely attributable to the mode of dexmedetomidine administration rather than variations in baseline comorbidities. In a similar study conducted by Niyogi et al., it was reported that none of the patients in both groups in their study had clinically significant tachycardia or hypertension during the study period [9].

The ASA classification system is a widely accepted tool for assessing the overall health status of patients before surgery, with higher ASA grades indicating a greater degree of systemic disease. The comparison of ASA grades between patients receiving intravenously administered dexmedetomidine (Group IV) and nebulized dexmedetomidine (Group IN) in this study offers a glimpse into the baseline health status of the study population. The predominance of ASA grade 1 patients in both groups suggests a cohort with minimal or no systemic disease, consistent with the aim of achieving comparable baseline characteristics. Singh et al. [16] and Niyogi et al. [9] have included 120 patients and 70 patients, respectively, of ASA I and II, but they have not shown a comparison of the distribution of patients between both groups. Clinically, the distribution of ASA grades is relevant for anesthesiologists in tailoring anesthetic management. Patients with higher ASA grades may be more prone to hemodynamic instability during airway interventions, and understanding the baseline health status aids in risk stratification and preoperative planning.

The comparison of baseline vitals between both groups is a critical aspect of understanding the potential physiological impact of different administration routes. The absence of significant differences in heart rate, systolic and diastolic BP, and the MAP between the two groups aligns with the observations in a study by Niyogi et al. [9]. This study investigated the hemodynamic effects of intravenous and nebulized dexmedetomidine in a similar population, emphasizing the comparable hemodynamic stability achieved with both administration routes.

The temporal changes in vital signs during and after induction provide crucial insights into the hemodynamic effects of intravenously (Group IV) and nebulized (Group IN) dexmedetomidine administration. The observed non-significant differences in heart rate at baseline, 0, 5, and 10 minutes after induction align with the findings of a study by Singh et al. [15], which investigated the immediate effects of dexmedetomidine on heart rate during induction. The significant decrease at 15 and 20 minutes after induction in Group IV compared to Group IN may be attributed to the prolonged and sustained impact of intravenous dexmedetomidine on the central nervous system, as also discussed by Singh et al. [16]. The significant reduction in systolic BP at 20 minutes in Group IV compared to Group IN indicates that intravenous administration of dexmedetomidine can lead to hypotension. The delayed but significant difference at 20 minutes underscores the importance of continuous monitoring during the post-induction period and suggests that intravenous dexmedetomidine may exert a more profound hypotensive effect than nebulized administration. The significant reduction in MAP at 15 minutes and 20 minutes in Group IV compared to Group IN corresponds with the findings of Niyogi et al. [9]. Moreover, in their study, it was seen that in the IV group, BP was slightly lower than the IN group at all time intervals [9]. The sustained impact at 15 and 20 minutes in the current study reinforces the notion of a prolonged hemodynamic effect of intravenous dexmedetomidine. The findings of our study also include a consistent and statistically significant reduction in heart rate, systolic and diastolic BPs, and MAP in Group IV compared to Group IN at various post-induction time points, which also emphasizes the need for vigilant monitoring, particularly during the post-induction period.

Until now, intravenous dexmedetomidine is most popularly used as premedication. However, it is documented that the sedative effect of IV dexmedetomidine is more pronounced than the analgesic effect, accompanied by profound bradycardia and hypotension. Moreover, IV dexmedetomidine may induce biphasic MAP oscillations, which are undesirable in anesthesia [17].

The statistically significant difference in median sedation scores between patients receiving nebulized dexmedetomidine (Group IN) and intravenously administered dexmedetomidine (Group IV) is a key observation that has implications for the assessment of sedation quality during the perioperative period. Sedation scores are essential indicators of a patient's level of consciousness and comfort during anesthesia and procedures. The lower median sedation score in Group IN, compared to Group IV, suggests that intravenous dexmedetomidine achieved a deeper level of sedation during the studied period. Similarly, Ankita et al. in their study reported that preoperative sedation was substantially greater in Group IV than in Group IN. The main disadvantage of IV dexmedetomidine is that sedative action is more pronounced than analgesic effect with profound bradycardia and hypotension [18]. Moreover, rapid IV dexmedetomidine infusion may cause biphasic alteration of MAP, which is undesirable in anesthesia [17, 19]. To minimize these adverse effects, alternative routes of dexmedetomidine were under trial.

Lakshmi et al. found that intranasal dexmedetomidine is a better premedication agent in morbidly obese patients than oral alprazolam [20]. Intranasal administration is more convenient because it is innocuous, odorless, and requires no intravenous infusion. Intranasal administration of a substance allows it to cross the blood-brain barrier and reach the central nervous system directly [21]. Because of the increased vascularity of the nasal mucosa, medications can gain rapid access to the venous blood of the systemic circulation, thereby bypassing first-pass metabolism in the liver [22].

There were some clinical studies where intranasal dexmedetomidine was administered as a premedication, especially in pediatric patients. In a study, Yuen et al. conducted a randomized comparison of two intranasal dexmedetomidine doses (1 μg/kg and 2 μg/kg) as premedication in children. They concluded that both doses produced a similar level of satisfactory sedation in children with no adverse hemodynamic effects in any of the groups [23]. It was proved that intranasal administration of dexmedetomidine is more effective at inducing sleep and a useful alternative premedication in children [24]. Intranasal dexmedetomidine also provides a reliable and effective method of providing sedation during CT scans [25].

In adult patients, the efficacy of IN dexmedetomidine has also been proved during both local and general anesthesia [26]. In a recent study by Hrishi et al., it has been well proved that IN dexmedetomidine (1μg/kg) provides good surgical field conditions along with the added advantages of lesser hemodynamic fluctuation during transnasal transphenoidal skull base surgery [27]. There were no statistically significant variations in heart rate and BP with a reduced anesthetic requirement in the IN dexmedetomidine group. IN dexmedetomidine also plays a considerable role in attenuating the increase in MAP during intubation response.

In another study by Wang et al., it was concluded that IN dexmedetomidine (1μg/kg) provides a considerable effect to attenuate the increase in MAP caused by intubation response. Changes in heart rate (HR) and Bispectral Index (BIS) also demonstrate that this kind of premedication provides effective attenuation of intubation responses [28].

Our study contributes to the understanding of the perioperative effects of different modes of dexmedetomidine administration, providing valuable insights for anesthesiologists in tailoring anesthesia management based on patient characteristics and procedural requirements. The safety and efficacy of nebulized dexmedetomidine in achieving optimal sedation and minimizing hemodynamic fluctuations warrant further exploration in diverse clinical settings and patient populations. The findings open avenues for future research, including larger-scale studies to confirm and generalize the results, as well as investigations into the safety and efficacy of nebulized dexmedetomidine in various clinical scenarios.

Limitation of study

Our study has certain limitations. We evaluated a single dose of dexmedetomidine, administered through intravenous and nebulized routes. We did not include other routes of administration, viz., intranasal route. Though we designed the study in such a way as to avoid any observer bias, we could not do any measurement of the uptake and distribution of dexmedetomidine administered by two different routes.

## Conclusions

In our study, the hemodynamic responses following laryngoscopy and tracheal intubation were more favorable with nebulized dexmedetomidine compared to intravenously administered dexmedetomidine. Intravenous administration of dexmedetomidine resulted in a greater decrease in heart rate, systolic and diastolic BP, and MAP, both before and after the induction of anesthesia, when compared to the nebulized route of administration. The sedation score was lower with nebulized dexmedetomidine than with intravenous administration. We conclude that nebulized dexmedetomidine is superior to intravenous dexmedetomidine in obtunding and maintaining hemodynamic stability following laryngoscopy and tracheal intubation.
